# Unearthing *EGFR* Mutations and the Rewards of Persistence in Precision Oncology: Breaching the 10-Year Survival Barrier in Metastatic NSCLC With Active Disease

**DOI:** 10.1200/JGO.19.00357

**Published:** 2020-01-31

**Authors:** Pawan Kumar Singh, Rajender Kumar, Amanjit Bal, Nalini Gupta, Rakesh Kapoor, Kuruswamy Thurai Prasad, Navneet Singh

**Affiliations:** ^1^ Department of Pulmonary Medicine, Postgraduate Institute of Medical Education and Research, Chandigarh, India; ^2^ Department of Nuclear Medicine, Postgraduate Institute of Medical Education and Research, Chandigarh, India; ^3^ Department of Histopathology, Postgraduate Institute of Medical Education and Research, Chandigarh, India; ^4^ Cytology and Gynecological Pathology, Postgraduate Institute of Medical Education and Research, Chandigarh, India; ^5^ Department of Radiotherapy, Postgraduate Institute of Medical Education and Research, Chandigarh, India

## INTRODUCTION

Management of advanced/metastatic nonsquamous non–small-cell lung cancer (NSCLC) has undergone remarkable transformation in the recent past. With the advent of targeted therapy against specific genetic alterations and programmed death-1 (PD-1)/programmed death-ligand 1 (PD-L1) immune checkpoint inhibitors (ICIs), treatment algorithms have become increasingly complex. This complexity is highlighted in 2 patients we recently encountered in our lung cancer clinic. Consent for publication was obtained from both patients described in this Case Report.

## CASE 1

**FIG 1:**
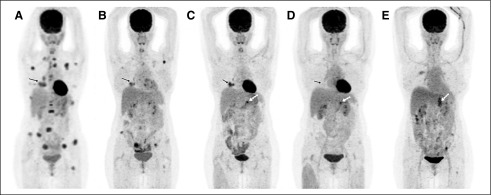
Maximum intensity projection images of (A) initial staging positron emission tomography (PET; August 2016) revealed an [^18^F]fluorodeoxyglucose (FDG)-avid soft tissue lesion in the right lower lobe (RLL; arrow) along with FDG-avid mediastinal/supraclavicular lymph nodes and multiple lytic bone lesions (appendicular and axial skeleton). (B) Follow-up PET (November 2016) depicted interval decrease in the size of the RLL lesion (arrow) and metabolic activity of the skeletal lesions. (C) Follow-up PET (March 2017) revealed an increase in metabolic activity of the RLL lesion (arrow) and appearance of a new FDG-avid left adrenal nodule (dashed white arrows). (D) Follow-up PET (September 2017) showed complete resolution of the RLL lesion and decrease in FDG avidity of the left adrenal nodule (dashed white arrow). (E) Follow-up PET (September 2018) revealed interval progression in the metabolic activity of the left adrenal nodule (dashed white arrow) and new-onset FDG-avid lesion in the left femur.

A 45-year-old woman who had never smoked, with no comorbidities, presented in August 2016 with a 2-month history of generalized body aches, fatigue, loss of appetite, and loss of weight. She was initially evaluated by a local physician and underwent a whole-body bone scan that revealed multiple lytic bony lesions. This prompted an [^18^F]fluorodeoxyglucose (18-FDG)–positron emission tomography (PET)/computed tomography (CT) scan, which revealed FDG-avid mediastinal/supraclavicular lymph nodes, lytic bone lesions (appendicular/axial skeleton), a soft tissue lesion in the right lower lobe (RLL), and bilateral random lung nodules (Fig 1A). Subsequently, she was referred to our clinic.

PET-guided biopsy from the vertebral lesion confirmed the presence of metastatic adenocarcinoma, whereas immunohistochemistry (IHC) showed strong nuclear positivity for thyroid transcription factor-1, suggesting lung origin. She was clinically staged as T4N3M1c (stage IVB; TNM classification, 8th edition). Molecular analysis was negative for epidermal growth factor receptor (*EGFR*) mutations (*EGFR*m) by real-time-polymerase chain reaction (RT-PCR), anaplastic lymphoma kinase (*ALK*; D5F3 IHC), and *ROS1* fluorescence in situ hybridization (FISH) rearrangements. Pemetrexed-carboplatin in standard dosages (every 3 weeks) and zoledronate (every 4 weeks) were started. Repeat imaging after 4 cycles showed partial response (PR; RECIST; Fig 1B). The patient received 2 additional cycles of platinum doublet, followed by maintenance pemetrexed (mPEM). Repeat imaging after cycle 2 of mPEM showed disease progression (PD; Fig 1C). At this stage, NGS (next-generation sequencing) on liquid biopsy was performed to look for any other potentially targetable genetic alterations that revealed exon 19 deletion (exon19del) *EGFR*m and led to initiation of afatinib 40 mg once daily. Grade 2 skin rash from afatinib mandated dose reduction to 40 mg/30 mg alternate days.

**FIG 2:**
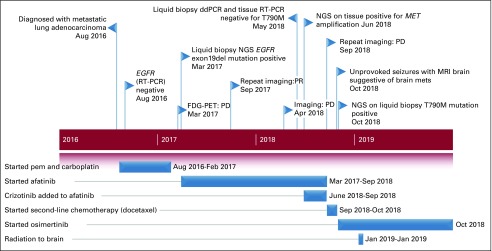
Timeline (case 1) of diagnosis, treatment received, response achieved, and molecular testing (dates and respective durations). Molecular testing methods (real-time polymerase chain reaction [RT-PCR], droplet digital polymerase chain reaction [ddPCR], next-generation sequencing [NGS]) listed are for detecting epidermal growth factor receptor (*EGFR*) mutations only. Exon19del, exon 19 deletion; FDG-PET, [^18^F]fluorodeoxyglucose–positron emission tomography; mets, metastases; MRI, magnetic resonance imaging; pem, pemetrexed; PD, progressive disease; PR, partial response.

Clinical and radiologic benefit (Fig 1D) was sustained for 15 months, but then PD was documented for both bony and adrenal lesions (Fig 1E). Repeat liquid biopsy (droplet digital PCR [ddPCR]) detected exon19del but no exon 20 T790M *EGFR*m. PET-guided biopsy from the adrenal gland confirmed metastatic adenocarcinoma. RT-PCR on rebiopsy was similar to ddPCR liquid biopsy (presence of exon19del but no T790M). To search for alternate mechanisms for acquired *EGFR* tyrosine kinase inhibitor (TKI) resistance, FISH showed *MET* amplification. Crizotinib was added to afatinib for targeting the latter. However, the patient tolerated dual-TKI combination poorly (grade 3 fatigue and nausea), requiring frequent dose reductions. Within 3 months, clinical and radiologic PD occurred, and both TKIs were discontinued. Second-line chemotherapy (docetaxel 75 mg/m^2^ every 3 weeks) was started. After 2 cycles, the patient developed generalized tonic-clonic seizures. Magnetic resonance imaging of the brain revealed multiple brain metastases, with surrounding edema. Antiepileptics and dexamethasone were started, and palliative whole-brain radiotherapy(8 Gy; single fraction) was given. The patient was unwilling to receive additional chemotherapy but willing to consider salvage treatment with PD-1/PD-L1 ICIs. PD-L1 testing (SP263 IHC) on archival tissue showed no expression (0%). The patient then opted for repeat NGS testing on liquid biopsy, which revealed the presence of both exon19del and T790M (mutation loads of 3.1% and 1.7%, respectively). Osimertinib was started (Fig 2) and led to substantial clinical benefit within 4-6 weeks. Currently, she is doing well, with follow-up imaging at 6 months showing PR.

## CASE 2

**FIG 3:**
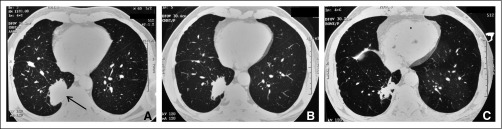
High-resolution computed tomography of the thorax at (A) baseline showing the right lower lobe (RLL) mass reaching up to the pleura. Multiple random nodules, some of which were subpleural, were also present in right lung, as was right-sided pleural effusion. (B) After 4 cycles of chemotherapy, showing reduction in the size of the RLL mass, as well as the number and size of the lung nodules. (C) During erlotinib and bevacizumab treatment, showing partial response in the form of decrease in size of the RLL mass, as well as size and number of lung nodules.

A 61-year-old man who never smoked, with no comorbidities, presented in November 2009 with right-sided pleuritic chest pain. Contrast-enhanced CT of the thorax revealed a 5.4-×-4-cm mass in the RLL, with right pleural effusion (Fig 3A). CT-guided fine-needle aspiration cytology (FNAC) from the RLL mass and pleural fluid cytology both showed adenocarcinoma. In view of TNM stage IVA (T2bN0M1a, 7th edition; T3N0M1a, 8th edition), 3 weekly pemetrexed-cisplatin was started. Repeat imaging after 4 cycles showed PR (RECIST; Fig 3B), leading to administration of two additional cycles of platinum doublet, followed by mPEM. However, imaging after cycle 2 of mPEM showed PD (new FDG-avid lesions in the right neck of the femur and subcarinal lymphadenopathy). The patient was started on erlotinib (150 mg by mouth once daily) and bevacizumab (7.5 mg/kg q3wkly), along with zoledronate.

**FIG 4:**
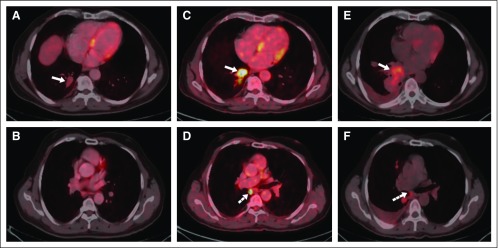
Transaxial fused [^18^F]fluorodeoxyglucose (FDG) positron emission tomography–computed tomography images. (A and B) After 3 years of erlotinib treatment (May 2013), a 2.6-×-1.7-cm FDG-avid nodule (maximum standardized uptake value [SUVmax], 2.7) in the right lower lobe (RLL; arrow), non–FDG-avid subcentimeter subpleural nodules, and non–FDG-avid right pleural thickening were visible. (C and D) After 6 years and 3 months of receiving erlotinib treatment (August 2016), an interval increase in metabolic activity of the RLL nodule (arrow; SUV max, 8.0) and subcarinal lymph node (dashed arrow; SUV max, 9.8) were visible. (E and F) In February 2018, an interval increase in size and metabolic activity of the RLL mass (arrow), with right subcarinal lymph node (dashed arrow) and reappearance of right pleural effusion, were visible.

Repeat imaging after 6 months showed PR (Fig 3C). Dual erlotinib-bevacizumab treatment was continued. However, the patient chose to discontinue bevacizumab after 9 cycles while erlotinib was continued. He was largely asymptomatic and stable clinically and radiologically for the next 6 years (with imaging being repeated every 6-12 months; Figs 4A and 4B). Although clinically stable while receiving treatment and with facilities for *EGFR*m testing becoming available at this time, liquid biopsy (ddPCR) was performed because there was no tissue in the initial diagnostic specimen (FNAC). However, results did not show the presence of any *EGFR*m. In the 7th year of erlotinib treatment, radiologic PD was documented (Figs 4C and 4D). However, being asymptomatic and because endobronchial ultrasound-guided transbronchial needle aspiration from the subcarinal lymph node was negative, the patient opted to continue erlotinib.

**FIG 5:**
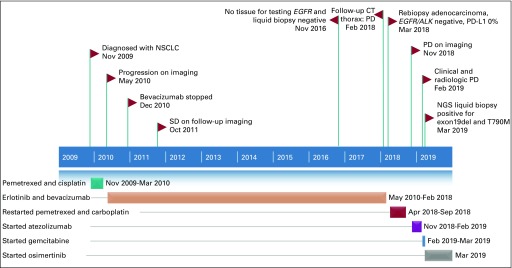
Timeline (case 2) of diagnosis, treatment, response, and duration, along with molecular testing. *ALK*, anaplastic lymphoma kinase; CT, computed tomography; *EGFR*, epidermal growth factor receptor; exon19del, exon 19 deletion; NGS, next-generation sequencing; NSCLC, non–small-cell lung cancer; PD, progressive disease; PD-L1, programmed death-ligand 1; SD, stable disease.

In the 9th year after diagnosis and while continuing to take erlotinib, the patient developed increased cough, breathlessness, anorexia, and weight loss. Repeat imaging (Figs 4E and 4F) showed an increase in the size of the RLL mass and right pleural effusion. Rebiopsy was performed to rule out histologic transformation and to obtain tissue for molecular analysis. Adenocarcinoma (lepidic pattern) was observed while testing for *EGFR*m (RT-PCR), *ALK*, and *ROS1* rearrangements (by D5F3 IHC and FISH, respectively), and *BRAF*m (sequencing) and *MET* amplification (FISH) were negative. With no evidence of any targetable genetic alteration, standard 3 weekly pemetrexed-carboplatin was started. After 4 cycles, repeat imaging showed PR and mPEM, and local thoracic radiation was given. Repeat imaging after cycle 4 of mPEM showed PD (increase in primary lesion and pleural effusion). Atezolizumab was started, but PD occurred after 5 cycles, and subsequently, single-agent gemcitabine was initiated, with stable disease being documented after 4 cycles. The patient declined additional chemotherapy. At this point, NGS was requested on liquid biopsy (because the previous tissue had been exhausted from prior processing) to look for any other targetable genetic alterations. NGS revealed the presence of classic exon19del, along with T790M (mutation load, 0.33%). Osimertinib was started (Fig 5), with symptomatic benefit within 4 weeks of treatment initiation. The patient has been observed for past 6 months and continues to derive clinical and radiologic benefit from this third-generation *EGFR* TKI.

## DISCUSSION

*EGFR* mutations are the most common type of targetable genetic aberrations in advanced/metastatic NSCLC. In our center, *EGFR*m prevalence varies from 40% in female nonsmokers to 11% in male smokers.^1^ The most common method for *EGFR*m testing is RT-PCR on tissue where a specific number of probes are used for the diagnosis of the most common type of *EGFR*m (exon19del and L858R insertion). After the initiation of therapy with first- and second-generation *EGFR* TKIs, patients invariably develop resistance, albeit after varying time periods. Mechanisms of resistance include acquired T790 exon 20 mutation (most common), histologic transformation to small-cell lung cancer, *MET* amplification, and so forth.^2^ The US Food and Drug Administration (FDA) has approved liquid biopsy testing by the ddPCR method for exon 20 T790M *EGFR*m.^3^ However, a negative liquid biopsy mandates tissue rebiopsy.

Furthermore, NGS in lung adenocarcinoma has emerged as a promising new tool to diagnose all targetable and nontargetable genetic alterations on a single platform. NGS can be applied on both liquid biopsy samples as well as tissue specimens.^4,5^

In both our patients, *EGFR*m was either not demonstrable or was proven to be negative by the currently recommended FDA-approved methods. But given the clinical profile of our patients (nonsmokers) and limited therapeutic options after conventional chemotherapy, repeated attempts were made to detect targetable mutations. Both the International Association for the Study of Lung Cancer and ASCO, in collaboration with other groups, have issued recommendations that multiplexed genetic sequencing panels are preferred over multiple single-gene tests to identify treatment options beyond the first-line testing.^6,7^

Case 1 demonstrated 3 important learning points:

1. Testing for *EGFR*m on a specimen obtained from bone can be falsely negative because of processing and decalcifying methods of the biopsy sample.
2. Demonstration of *MET* amplification (after T790M testing is negative) can provide an opportunity for dual-TKI therapy. However, the significance as well as clinical benefit of dual-TKI therapy remains unknown.^8,9^
3. In cases where clinical suspicion of *EGFR*m is high, repeated attempts for testing, using various samples (tissue/blood) and using various methods (RT-PCR, ddPCR, NGS) may be worthwhile.

Case 2 demonstrated the following:

1. The utility of NGS in blood after proven negativity for *EGFR*m in biopsy specimen. Tumor heterogeneity is one of the proposed mechanisms for inability to demonstrate *EGFR*m in tissue postprogression.^10^
2. Survival beyond 10 years after diagnosis with stage IV NSCLC. The patient’s treatment was started in an era when *EGFR*m testing was not freely available in our country, and erlotinib was approved for treatment of relapsed NSCLC (without knowing *EGFR*m status).^11-14^ Subsequently, based on the patient’s clinical profile and with availability of newer molecular testing platforms, multiple testing methods (RT-PCR, ddPCR, NGS) were used to unravel the mystery of unexpected and sustained clinical benefit with erlotinib, as well as 10-year survival with metastatic disease.
3. Lack of benefit with PD-L1 ICIs is consistent with emerging data about lack of efficacy of PD-1/PD-L1 ICIs in patients with *EGFR*m, especially those with acquired T790M.^15-17^

Metastatic lung adenocarcinoma management in the era of precision oncology offers opportunities for the astute clinician to use increasingly sensitive molecular diagnostic methods in an attempt to offer the most appropriate targeted therapy at each time point in the patient’s disease. The results of such an approach are often rewarding both for the clinician and the patient/caregivers, and the previous concept of nihilism for stage IV NSCLC clearly needs to be abandoned now, even in emerging economies with resource constraints.
